# Clinical Society of Maryland

**Published:** 1894-08

**Authors:** H. O. Reik

**Affiliations:** Secretary; 525 N. Howard St.; Baltimore


### CLINICAL SOCIETY OF MARYLAND.

BALTIMORE, April 20, 1894.

The 295th regular meeting of the Society was called to order by the President, DR. J. EDWIN MICHAEL.

DR. W. E. MOSELEY read a paper on the "Importance of a Microscopical Examination in the Differential Diagnosis of Diseases of the Uterus," treating the subject from a clinical standpoint.

DR. H. HARLAN read a paper on "Cases of Foreign Bodies in the Eye, associated with Sympathetic Ophthalmia."

The first was that of a young man, aged thirty-seven, who had received a blow in the eye from a piece of the head of a nail fifteen years previously. He consulted me because the good eye had recently been growing misty. Ophthalmoscopic examination showed iridoplegia and slight neuritis with the dimness of vision, and regarding the trouble as of sympathetic origin, I advised enucleation of the lost eye. The bit of steel was found just posterior to the ciliary bodies.

Case second was a farmer aged thirty-eight who came with the right eye red and inflamed, without light perception and painful to the touch. History was, that seven years previous while passing through a woods he had been struck in the eye by a branch of a sweet gum tree. Eye has been more or less painful ever since, at times very painful. The sound eye has never given any trouble. After enucleation the eyeball was opened and just behind the iris was found an entire small terminal bud, conical in shape and covered by the hard shell that is found over the buds of sweet gum in late winter.

Here we have two cases where a foreign substance remained in an eye many years, one fifteen and the other seven. One a particle of steel which possibly entered in an aseptic condition. The other a fresh growing vegetable fiber. One remaining quiescent for fifteen years and then setting up sympathetic trouble in the good eye; the other probably carrying many organisms and causing nearly constant irritation for seven years and causing no trouble whatever in the sound eye.

Dr. Harlan then considered at some length the history of sympathetic ophthalmia, its causes and the various theories as to how it is brought about. He was rather inclined to take issue with Deutschman and to support the ciliary nerve theory, concluding as follows:

"We must admit that notwithstanding a great deal of well directed experimental work, little has been accomplished towards the solution of the problem of sympathetic ophthalmia. The fact that its infectious origin has not yet been clearly proven is to my mind one of the strongest arguments against its being of that nature. If infectious, micro-organisms ought to be present always and frequently found in eyes taken out for causing sympathetic trouble in the fellow eye. Again, if infectious, by whatever means the infection spreads, whether by way of nerves, blood-vessels or lymph channels, there is no satisfactory explanation of the disease being so strictly a local one.

DR. HIRAM WOODS said, that the nature of sympathetic ophthalmia seemed as mysterious as ever. He did not think that Deutschman's explanation had been completely overthrown. His researches, as well as those of Alt and Gifford, had proved that infection of one can involve the other eye; that the path lies most probably along the lymph channels of the optic nerve, the orbital vessels and sub-orbital space. Negative cannot always set aside positive testimony. In this instance it has proved that infection of one does not necessarily involve the other eye; possibly does so only rarely. The well known frequency of sympathetic ophthalmia after panophthalmitis is a bit of clinical evidence against the liability of an eye to become inflamed when its fellow is suffering from an infection. Dr. R. L. Randolph was quoted as authority for the statement that organisms are rarely found in an eye enucleated after sympathetic disease has started in the other. All this proves that there must be some other explanation than infection; but does not disprove the possibility of the latter against well authenticated positive results.

Dr. Woods narrated two cases of sympathetic ophthalmia following injuries which did not destroy sight. In one patient both eyes were ultimately destroyed by the plastic irido-cyclitis, light perception alone remaining. In the other the injured eye eventually retained better vision than the other.

H. O. REIK, M.D., Secretary.

525 *N. Howard St.*

---

### THE FORMS OF DIABETES.

Dr. George Harley gives the following classification of diabetes

1. Hepatic diabetes—including the gouty variety.

2. Cerebral diabetes—including all cases of saccharine urine arising from nerve derangements.

3. Pancreatic diabetes—the most deadly form of the disease.

4. Hereditary diabetes—a form by no means uncommon, and one, too, where both brothers and sisters may labor under the disease without either their maternal or paternal parent having been affected by diabetes, though more distant members of the family may have suffered from it.

5. Food diabetes—including all forms of saccharine urine arising from the ingestion of unwholesome substance.

In the matter of treatment, besides diet and opium or codeine, Dr. Harley recommends croton chloral, strychnine, phosphoric acid for thirst, and an absolute prohibition of alcohol.—*Med. Record.*

---

### GOITRE.

Dr. Meusel (*La Semaine Médicale*, No. 1, 1894) regards the most efficacious medical treatment of goitre to consist in the internal administration of digitalis and local application of a salve of the iodide of potash to the growth. Under the influence of this treatment he has caused goitres to diminish in size, and even to disappear with astonishing rapidity, which have resisted treatment by the iodides, both internally and externally. The favorable action of digitalis in hypertrophy of thyroid is explainable by its influence on arterial tension. This employment of digitalis is far from being new, for Dr. Murray, in 1776, used it in this affection.—*Ex.*

